# Ultrashort Pulse Excited Tip-Enhanced Raman Spectroscopy in Molecules

**DOI:** 10.1021/acs.nanolett.2c00485

**Published:** 2022-06-15

**Authors:** Yang Luo, Alberto Martin-Jimenez, Rico Gutzler, Manish Garg, Klaus Kern

**Affiliations:** † Max Planck Institute for Solid State Research, Heisenbergstrasse 1, 70569 Stuttgart, Germany; ‡ Institut de Physique, Ecole Polytechnique Fédérale de Lausanne, 1015 Lausanne, Switzerland

**Keywords:** Tip-enhanced Raman spectroscopy, ultrafast Raman spectroscopy, femtosecond pulses, STM, molecular vibrations

## Abstract

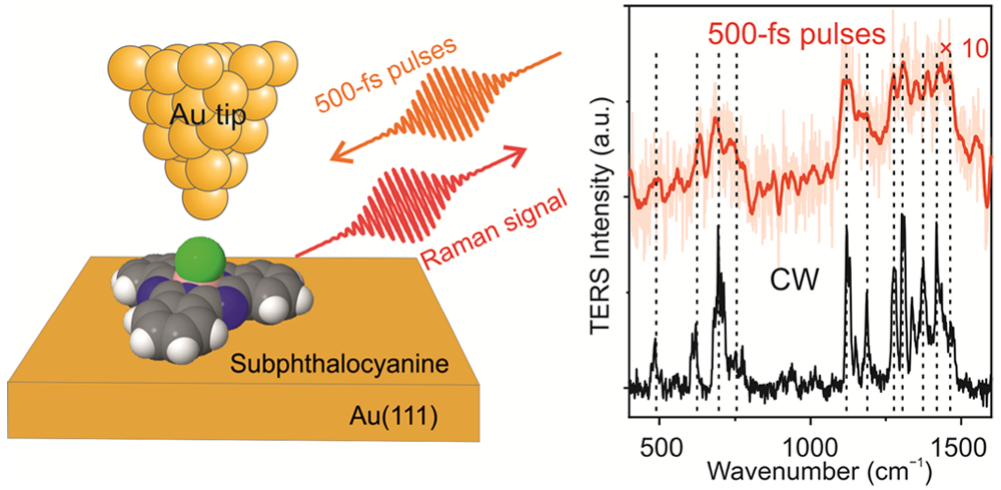

Vibrational fingerprints of molecules and low-dimension materials can be traced with subnanometer resolution by performing Tip-enhanced Raman spectroscopy (TERS) in a scanning tunneling microscope (STM). Strong atomic-scale localization of light in the plasmonic nanocavity of the STM enables high spatial resolution in STM-TERS; however, the temporal resolution is so far limited. Here, we demonstrate stable TERS measurements from subphthalocyanine (SubPc) molecules excited by ∼500 fs long laser pulses in a low-temperature (LT) ultrahigh-vacuum (UHV) STM. The intensity of the TERS signal excited with ultrashort pulses scales linearly with the increasing flux of the laser pulses and exponentially with the decreasing gap-size of the plasmonic nanocavity. Furthermore, we compare the characteristic features of TERS excited with ultrashort pulses and with a continuous-wave (CW) laser. Our work lays the foundation for future experiments of time-resolved femtosecond TERS for the investigation of molecular dynamics with utmost spatial, temporal, and energy resolutions simultaneously.

Many physical and chemical processes of key fundamental importance evolve on extremely fast time scales (attosecond to picoseconds) and at very short length scales (picometer to nanometer).^1−3^ Understanding the ultrafast dynamics at the nanometer scale is the key to the development of novel optoelectronic and nanoelectronic devices, as well as molecular scale study and control of chemical transformations. By combining ultrafast lasers with an STM, experiments have now demonstrated the capability to probe electron and charge dynamics at the nanoscale.^4−19^ While these experiments can provide information on electron and charge dynamics with subfemtosecond temporal and subnanometer spatial resolutions, they fail to provide information on the dynamics of the vibrational modes of a molecule or a semiconductor. Ultrafast experiments performed with an STM usually measure a time varying quantity from which the electron motion can be inferred, while fail to monitor the dynamics of vibrational modes and its coupling with electron dynamics. An extensively explored strategy to spectrally trace vibrational modes with subnanometer spatial resolution is tip-enhanced Raman spectroscopy (TERS),^20−41^ which unifies the advantages of Raman spectroscopy and the spatial resolving capability of scanning probe microscopy (SPM). Performed in a low-temperature ultrahigh-vacuum STM, TERS experiments have demonstrated the capability to capture the spatial distribution of vibrational modes of a single molecule with sub molecular resolution.^34,35^ Nevertheless, the temporal resolution in STM-TERS excited with continuous-wave (CW) laser is usually limited to the time scale of seconds, which is too slow to probe vibrational coherences and dynamics in molecules, which evolve on much faster time scales (fs to ps). The first step to develop ultrafast TERS techniques in an STM for probing vibrational dynamics with femtosecond temporal and subnanometer spatial resolutions is the measurement of TERS signal excited with ultrashort laser pulses.

In earlier attempts to measure TERS with ultrashort laser pulses in an STM, Van Duyne’s group conducted experiments to obtain TERS signal from molecules excited with few ps long laser pulses.^42,43^ They have reported a degradation of the TERS signal, possibly because of an unstable plasmonic cavity, reactive decay, or molecular diffusion.^42^ The signal decay behavior was also observed under UHV conditions,^43^ which could in principle suppress both diffusive and reactive processes. Therefore, it is desirable to establish optimal conditions for performing ultrafast-TERS experiments. Moreover, a few picoseconds temporal resolution is still insufficient for the study of vibrational coherence and dynamics in molecules; thus, time-resolved TERS with femtosecond laser pulses is demanded to achieve the stated goal.

In this work, we demonstrate ultrafast TERS excited by ultrashort laser pulses, which are ∼500 fs long, in a LT-UHV-STM. Distinct vibrational (Raman) features were measured from subphthalocyanine (SubPc) molecules on top of Au(111). The spectral resolution in the Raman spectra measured by ultrashort laser pulses is inversely related to their temporal resolution; TERS spectra measured with ∼500 fs long laser pulses provide an optimal balance between spectral and temporal resolutions. We investigate here the stability of the TERS signal over time and its dependence on the flux of the incident laser pulses and the plasmonic (tunneling) gap size. Furthermore, detailed comparisons of the TERS signal obtained with CW excitation and with ultrashort pulse excitation on the aforementioned aspects are presented.

Our experiments were performed in a home-built side-illumination TERS setup as shown in Figure 1a. The ultrafast laser system used in the current work is a Ti:sapphire oscillator (Element 2, Newport Spectra-Physics) which produces laser pulses of ∼6 fs duration with a bandwidth spanning from 650 to 1050 nm at a repetition rate of ∼80 MHz. Laser pulses centered at ∼728 nm and with a duration of ∼500 fs were generated by narrowband filtering (Ultra Narrow Bandpass Filter 728.1/1.5, AHF) of the broadband ∼6 fs long laser pulses. An achromatic lens (diameter = 50 mm; focusing length = 75 mm) is mounted inside the UHV chamber to focus the laser beam onto the apex of the Au tip. The TERS signal was collected through the same achromatic lens and then focused onto the entrance slit of a spectrometer (Kymera 328i, ANDOR) and detected by a thermoelectrically cooled charge coupled device (iDus 416, ANDOR). TERS experiments were also performed with CW excitation by using a helium–neon (He–Ne) CW laser (HNL150L, Thorlabs) centered at ∼633 nm.

**Figure 1:**
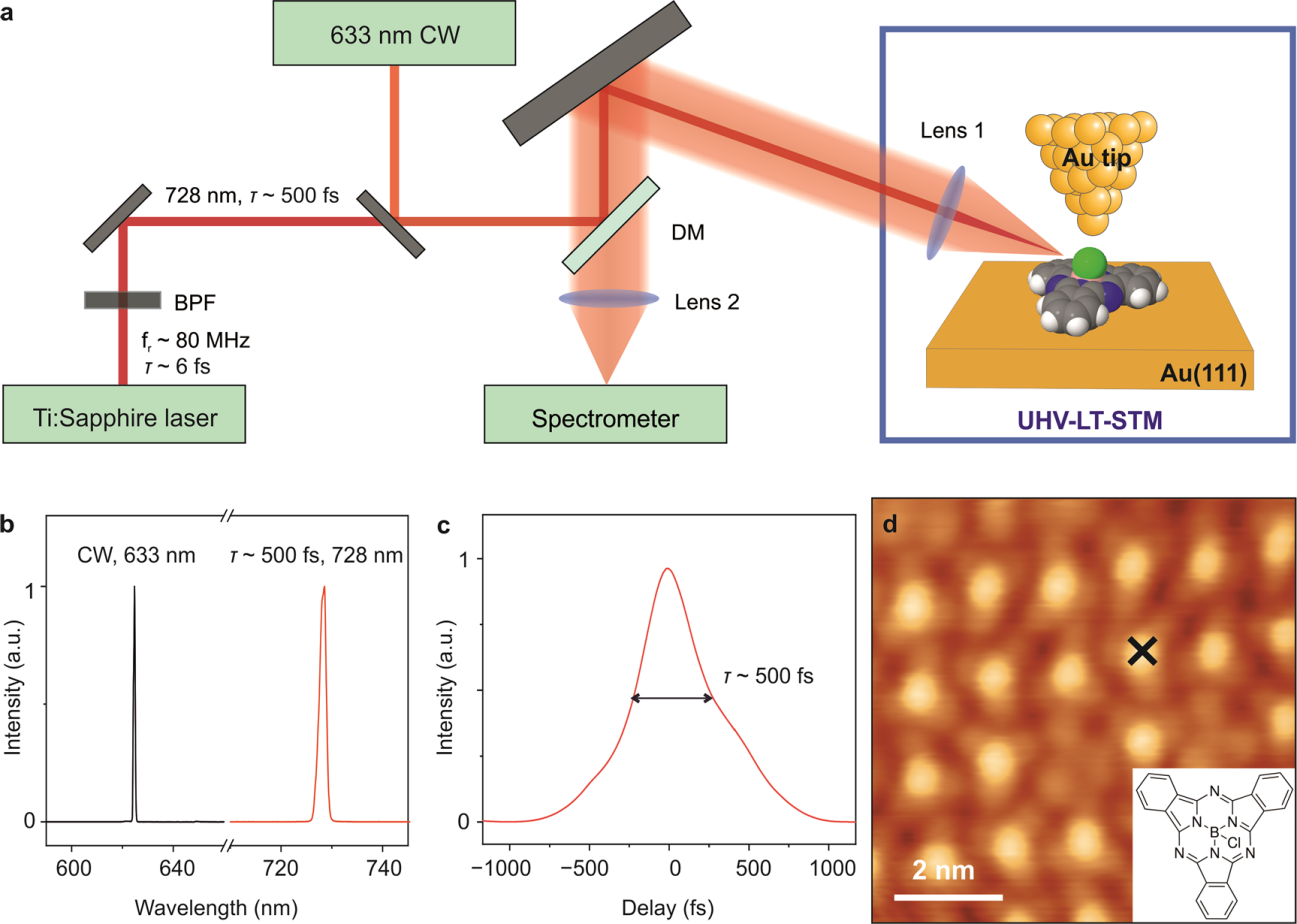
(a) Schematic illustration of the experimental setup. BPF: Bandpass filter. DM: Dichroic mirror. (b) Experimentally measured spectrum of the CW laser at 633 nm (black curve) and ultrashort pulses (red curve, duration τ ∼ 500 fs, centered at 728 nm) at the STM junction. (c) Temporal intensity profile of the ultrashort pulses locally characterized at the STM tunnel junction. Ultrashort pulses were characterized by the technique of homodyne beating. (d) STM image of one-monolayer of SubPc molecules adsorbed on Au(111) measured at a bias voltage of *V* = 1 V and a set-point current of *I* = 20 pA.

The spectral resolution of our TERS setup was determined by measuring the spectrum of the scattered laser light from the STM junction. Figure 1b shows the laser spectra of the CW beam centered at ∼633 nm and the ultrashort pulses centered at ∼728 nm. The full-width-half-maximum (fwhm) of the CW laser is around 10 cm^–1^, which is primarily influenced by the instrumental resolution resulting from the slit size of the spectrometer and the focal length of the lenses used in the setup. A fwhm of ∼30 cm^–1^ is obtained for the spectrum of the ultrashort pulses, which is consistent with the ∼1.5 nm bandwidth of the ultranarrow bandpass filter. The duration of the ultrashort pulses was locally measured at the plasmonic junction by the technique of homodyne beating as demonstrated in our earlier publication (see also Figure S1).^15,16^ The temporal intensity profile of the ultrashort pulse as characterized by this technique is shown in Figure 1c. The measured duration of the laser pulse is approximately 500 fs, which is close to the minimum possible duration of a bandwidth-limited Gaussian pulse centered at 728 nm with a spectral bandwidth of ∼1.5 nm. Therefore, in the TERS measurements with ultrashort laser pulse excitation, the spectral resolution of the vibrational modes would be ∼30 cm^–1^ at best and the temporal resolution would be ∼500 fs. The local characterization of ultrashort pulses is crucial in order to truly estimate the temporal resolution of the setup.

In our measurements, the average laser power density is ∼50 W/cm^2^ at the STM junction for both the CW laser and ultrashort laser pulses. However, the duration of the ultrashort pulses is ∼500 fs, implying that the peak power density in the case of ultrafast pulses is ∼1 × 10^6^ W/cm^2^, which is ∼10^4^ times higher compared to the CW laser. The peak electric field of the laser pulses at the STM junction will be significantly higher than the free space electric field because of the local enhancement arising from the nanocavity plasmon. Such a strong electric field at the STM junction could result in laser-induced damage of the nanocavity and diffusion of molecules in ambient conditions.^42^ To minimize the undesirable diffusion or reactive processes, all our TERS experiments were carried out with a home-built STM operating in ultrahigh vacuum conditions (∼5 × 10^–10^ mbar), and at liquid nitrogen temperature (∼90K). Clean Au(111) surfaces were prepared by repeated cycles of sputtering with Ar^+^ ions, followed by thermal annealing at 500 °C. Electrochemically etched Au tips were used to couple the laser pulses into the STM junction and enhance the Raman signal by plasmonic enhancement. One monolayer (ML) of SubPc molecules were deposited on Au(111) by thermal sublimation from a resistively heated evaporator. We observe two different configurations of the SubPc molecules in the molecular monolayer as shown in Figures 1d and S2. The majority of the SubPc molecules, which present a bright protrusion in the center, are in the “chlorine-up” binding configuration, and the molecules showing three lobes (and no central bright protrusion) are in the “chlorine-down” configuration.^32^ We observe no difference in the TERS spectra when the STM tip is placed on top of the molecules where the Cl atom points up or when it points down as shown in Figure S3, which is most likely due to the lack of structural changes in the molecule in the two configurations. All TERS spectra presented in the current work were measured with the STM tip placed at the center of a molecule where the Cl atom points up. Switching between the two different adsorption geometries of the SubPc molecules on top of Au(111) was not observed with neither the CW beam nor ultrashort pulses. This is most likely due to a higher energy barrier between the two configurations of the SubPc molecule compared to the photon energy of the laser pulses (<2 eV).

A TERS spectrum measured with ∼500 fs pulse excitation is shown in Figure 2a. Multiple vibrational peaks in the energy range of 400–1600 cm^–1^ can be clearly identified in the TERS spectra measured with both CW excitation (black curve), as well as ∼500 fs pulse-excitation (red curve). The positions of the Raman peaks, denoted by vertical black dashed lines in Figure 2a, and their relative intensities are similar for both types of excitation schemes. Most of the vibrational peaks match quite good with the simulated Raman spectrum (blue curve) from a single SubPc molecule in absence of the Au(111) surface by density functional theory (DFT) calculations. Orca 4.2.1^44^ was used in the calculations with the B3LYP functional and the def2-SVP basis set,^45^ and the dispersion interaction is accounted for by the atom-pair wise dispersion correction.^46^ The spectral width of the Raman peaks in the TERS spectrum excited with ultrashort pulses is larger compared to the TERS spectrum excited with the CW laser due to the reduced spectral resolution, which is primarily decided by the spectral bandwidth of the ultrashort pulses (Figure 1b). It should be noted that in the TERS experiments with ultrashort pulse excitation, the temporal and spectral resolutions are inversely related. A high spectral resolution implies a lower temporal resolution and vice versa. In the current case of transform limited laser pulses centered at 728 nm with a bandwidth of 1.5 nm, we could obtain an energy resolution of Δν ≈ 30 cm^–1^ and a temporal resolution of Δ*t* ≈ 500 fs. By using a 3 nm bandpass fitter centered at the same wavelength, one could get an increased temporal resolution of ∼250 fs, with a reduced spectral resolution of ∼60 cm^–1^. By properly designing the laser pulses, we can balance the spectral and temporal resolutions in the time-resolved measurements. In the TERS measurements using ∼500 fs long laser pulses, we are able to distinguish all the strong Raman peaks at 480, 620, 700, 1120, 1190, 1280, 1310, 1370, and 1420 cm^–1^. Because of the reduced spectral resolution, the less strong vibrational peaks at 550, 940, 1015, 1150, 1480, and 1560 cm^–1^ are less obvious. Shorter pulses (e.g., ∼250 fs long) would significantly reduce the spectral resolution, hence blurring the features in the Raman spectrum.

**Figure 2:**
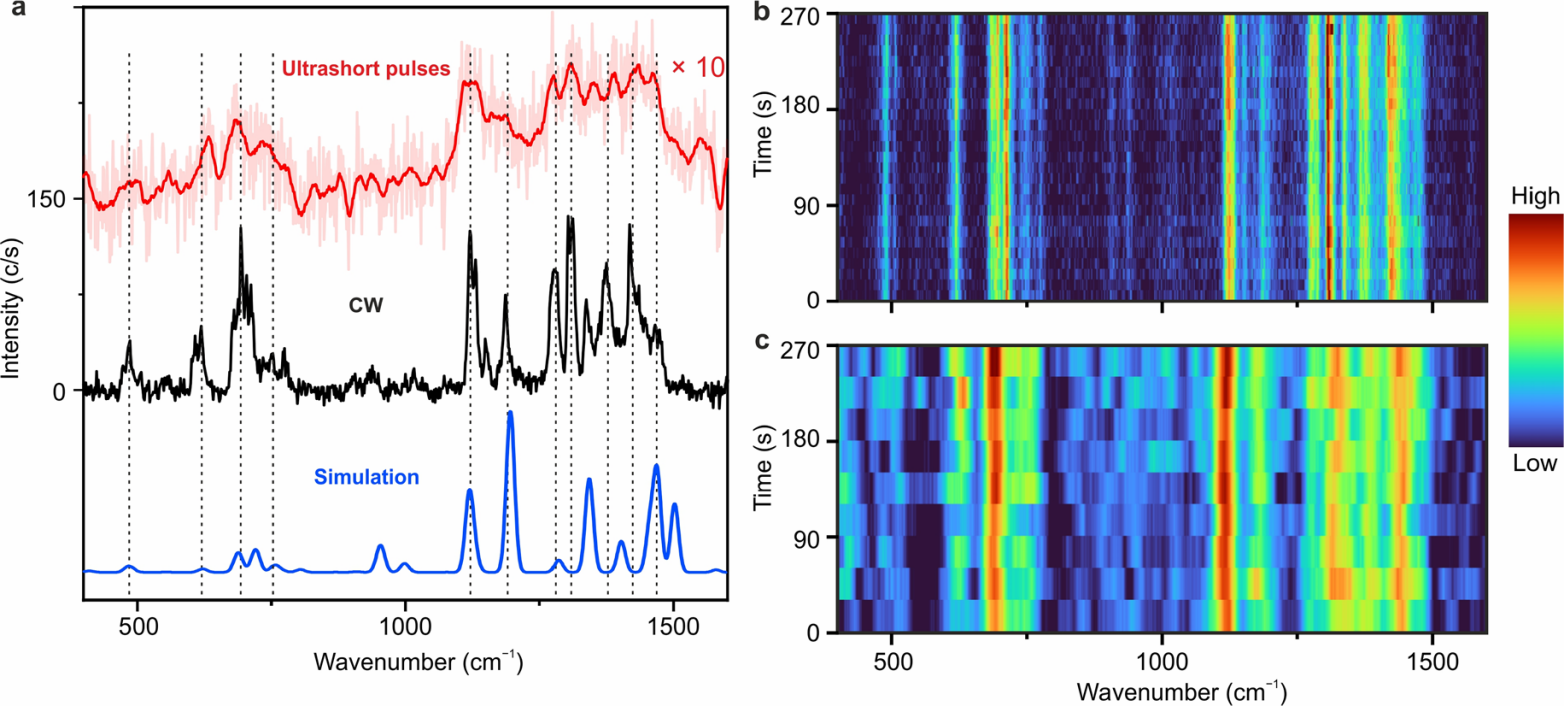
(a) Comparison of the TERS spectra acquired with CW excitation (black curve, λ = 633 nm, laser power *P* = 1.1 mW, bias at the STM junction *V* = 10 mV, set-current *I* = 1 nA, acquisition time *t* = 60 s), and with ∼500 fs long laser pulses (red curve, λ = 728 nm, *P* = 1.5 mW, *V* = 10 mV, *I* = 8 nA, *t* = 120 s). (b) Series of 27 consecutively measured CW-TERS spectra (*t* = 10 s). (c) Series of 9 consecutively measured TERS spectra with ∼500 fs laser pulse excitation (*t* = 30 s). The TERS spectra measured with ultrashort pulses were smoothed by Savitzky–Golay filtering. All TERS spectra are subtracted from the broad background of scattered light for clarity (Figure S4).

The spectral intensity of the Raman peaks in the TERS spectrum measured with ultrashort pulses is nearly an order of magnitude lower than when measured with CW excitation, due to several reasons. First, the spectral line width of the Raman peaks in the TERS spectrum measured with ultrashort pulses is ∼3 times larger compared to the line widths measured with CW excitation, leading to a lower peak height in the spectral intensity. Second, the relative position of the CW excitation laser at 633 nm and the ultrashort pulse excitation at 728 nm with respect to the plasmonic resonance of the STM nanocavity is different.^25,26,36^ A representative spectrum of the plasmonic emission (spectrum of resonance) at the STM junction can be seen in Figure S5. The Raman features appear close to the resonance position of the plasmonic emission for the case of the TERS spectrum measured with CW laser, however, not for the ultrashort pulses. Therefore, the enhancement factor for the CW-TERS would be higher, producing higher intensity of the Raman signal compared with TERS measured with the ultrashort pulses. To verify the role of plasmonic enhancement of the TERS signal, we varied the central wavelength of the ultrashort pulses from 723 to 696 nm using an acousto-optic-tunable-filter (AOTF) (Figure S6). A stronger Raman signal is measured when exciting with laser pulses with a central wavelength of 696 nm (Figure S7). Limited by the bandwidth of the few-cycle ultrashort laser pulses of our laser source, a direct comparison of the spectral intensity of CW-TERS and TERS measured with ultrashort pulses at the same excitation wavelength is not possible in the current work. Third, the focal spot size of the ultrashort pulses is slightly bigger compared with the CW beam due to a worse laser mode (not perfectly Gaussian).

The stability of the TERS signal was determined by the sequential measurement of TERS spectra over time. Figure 2b shows a series of 27 CW-TERS spectra, demonstrating the stability of the TERS signal with CW excitation over ∼270 s. Stable TERS signal over time was also observed for ultrashort pulse excitation (Figure 2c), even though the peak power of the ultrashort pulses is much higher compared to the CW beam. This is in contrast with previous results of STM-TERS with few ps excitation, which have shown signal decays caused by instability of the nanocavity or diffusion.^42,43^ Our results transparently show that molecules in a plasmonic junction (Au tip–SubPc molecule–Au substrate) of approximately one nanometer gap size remain stable in UHV and at cryogenic temperatures upon excitation with ultrashort laser pulses. The input power of the ultrashort laser pulses can in principle be even higher than 1.5 mW, as used in the current work. The energy of the ultrashort pulses generated in the current work is <25 pJ (2 mW). For laser pulses in this range, we do not observe any damage in the plasmonic junction of the STM. At much higher peak pulse energies, for example, >200 pJ, damage of the plasmonic junction may occur.

In a spontaneous Raman scattering process, the strength of Raman scattering (signal) is determined by the first order response of the induced polarization, *P*^(1)^ = ε_0_χ^(1)^*E*, where ε_0_ is the permittivity of free space, χ^(1)^ is the first-order susceptibility and *E* is the local electric field. Thus, the intensity of the Raman transition in the case of spontaneous emission is proportional to the incident laser power, *I*_Raman_ ∝ *I*_laser_. Figure 3a and 3c shows a series of measured TERS spectra with increasing incident laser power for the cases of CW and ultrashort pulse excitation, respectively. The variation of the integrated intensity of the TERS signal with increasing power of the CW laser and ultrashort pulses is shown in Figure 3b and 3d, respectively. A linear dependence of the intensity of the TERS signal with respect to the power of the incident laser has been observed in the case of CW-TERS (Figure 3b) as well as for the ultrashort pulse excited TERS (Figure 3d), as shown by the linear fitting (red curves) of the experimental data (black points). This suggests that the TERS signal purely arises from spontaneous Raman transitions, in which the incident photon interacts with the molecule and scatters inelastically. Although the photon flux for the ultrashort pulses is much higher compared to the CW laser, the TERS process is still in the linear regime.

**Figure 3:**
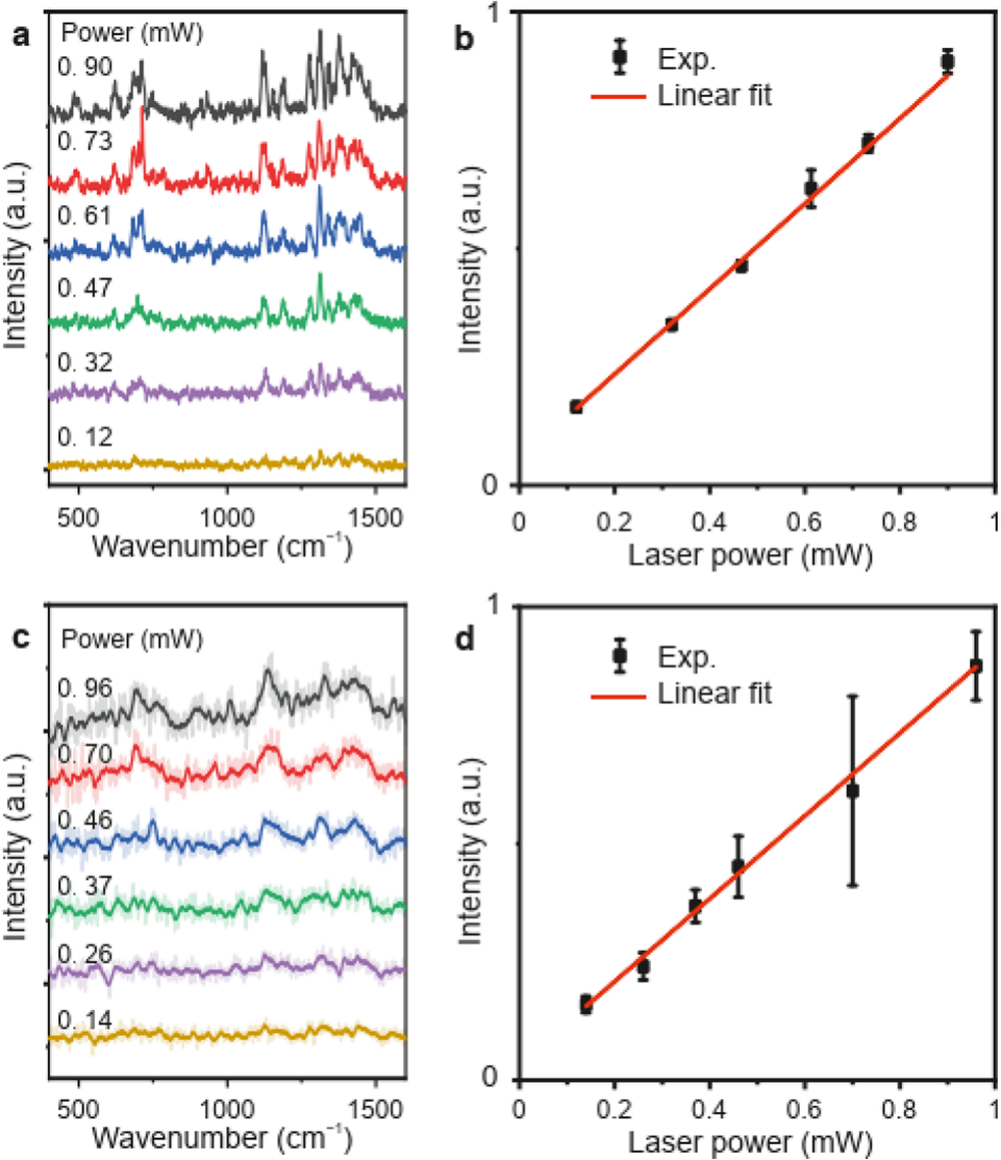
(a) Measured variation of the CW-TERS spectra as a function of the incident laser power in the range from 0.12 to 0.90 mW. The spectra were acquired with *V* = 10 mV, *I* = 8 nA, and with an acquisition time of 10 s. (b) Scaling of the overall TERS signal on increase of the power of the exciting CW laser. (c) Measured variation of the TERS spectra excited with ultrashort pulses as a function of the incident laser power in the range from 0.14 to 0.96 mW. (d) Scaling of the overall TERS signal on increase of the power of the exciting ultrashort laser pulses. The TERS intensities were calculated by integrating the area under all the Raman peaks. The spectra were acquired under STM conditions of *V* = 10 mV and *I* = 8 nA, with acquisition time of 30 s. The laser power used in the different measurements is annotated on top of each spectrum. Error bars in panels c and d represent the standard deviation of the signal from 5 consecutive measurements. The red-curve in panels b and d represents the linear fitting of the experimentally measured points (black squares).

The strong atomic localization of light in the nanocavity plasmonic junction formed by the sharp metallic tip and the metallic substrate is the key to a high lateral resolution in resolving the spatial distribution of distinct vibrational modes of molecules. The exponential dependence of the tunneling current with respect to the tip–sample distance in an STM gives rise to very high lateral resolution. Similarly, the highly nonlinear dependence of the intensity of the TERS signal on the size of the nanocavity determines the lateral resolution in TERS measurements. To assess the spatial resolution of TERS, we have measured the variation of the intensity of the TERS signal with the plasmonic gap size at the STM junction. Here, the size of the nanocavity is determined by the tip–sample distance, which can be varied and stabilized with picometer resolution in an STM. Figure 4a shows a series of TERS spectra measured with increasing plasmonic gap (decreasing tunneling current) size for the case of excitation with the CW beam. The plasmonic gap between the atomically sharp tip and the molecule can be carefully varied by modifying the tunneling current in the STM junction operating under constant current feedback conditions (Figure S8). The overall intensity of the TERS signal obtained by integrating the area under the Raman peaks in Figure 4a decays exponentially with increasing plasmonic gap size, as shown in Figure 4b. The variation of the intensity of the TERS signal can be fitted with an exponential function, *I*_TERS_ ∝ exp(−*d*/*k*), with a decay length of *k* = 150 pm, connoting to an exponential dependence of the TERS signal on the plasmonic gap size. Here, *d* refers to the relative variation of the plasmonic gap. The variation of the intensity of the TERS signal with respect to the plasmonic gap was also measured for excitation with ultrashort pulses (Figures 4c-4d). A similar decay length of ∼140 pm is obtained, suggesting a very similar dependence of the TERS signal on the plasmonic gap size. It is worth mentioning that in a nonideal scenario where a molecule jumps from the Au(111) surface and gets partially attached to the nanotip of the plasmonic junction, a much larger decay length of ∼400 pm was obtained as shown in Figure S9. The Raman signal from a contaminated tip can still be detected even when the STM tip is more than 2 nm away from the Au(111) surface.

**Figure 4:**
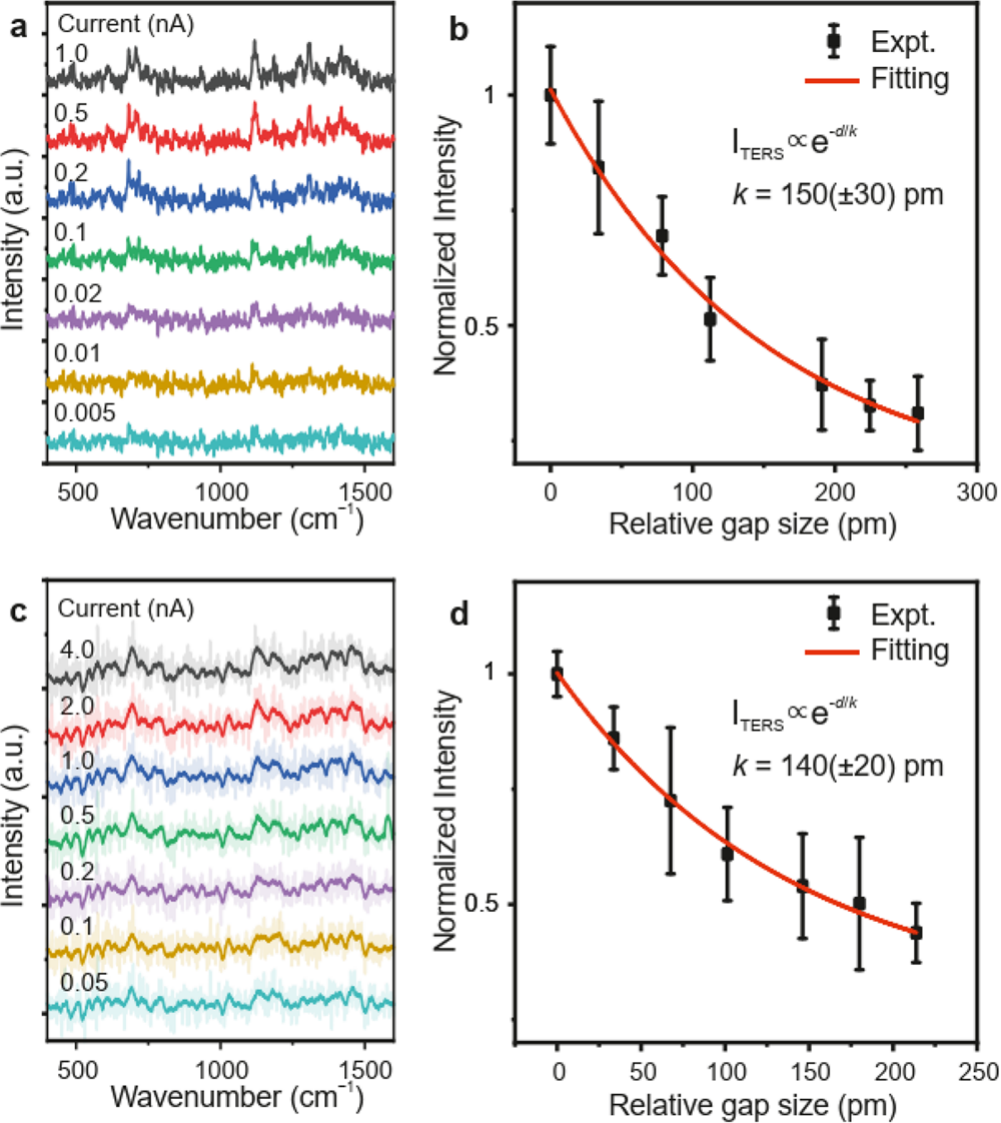
(a) Series of CW-TERS spectra acquired as a function of decreasing tunneling current (increasing plasmonic gap size) at the STM junction. The spectra were acquired with an incident laser power of 0.75 mW, with a bias voltage of 10 mV at the STM junction. The acquisition time of each spectrum was 10 s. (b) Variation of the integrated intensity of the CW-TERS signal with the relative plasmonic gap size. (c) Series of TERS spectra measured with ultrashort pulses as a function of decreasing tunneling current (increasing plasmonic gap size) at the STM junction. The spectra were acquired for an incident laser power of 0.57 mW, with a bias voltage of 10 mV at the STM junction. The acquisition time of each spectrum was 30 s. (d) Variation of the intensity of the TERS signal excited by the ultrashort pulses with the size of the plasmonic gap. The red curves in panels b and d represents the exponential fitting of the experimentally measured points (black points). Error bars in panels b and d represent the standard deviation of the signal from 5 consecutive measurements.

The obtained decay lengths of the TERS signal on change of the plasmonic gap size are similar to the values reported in other works,^26,30^ in which subnanometer lateral resolution of the TERS signal was demonstrated. Since our experiments were performed on a sample with full-monolayer coverage of SubPc molecules, we do not present here lateral spatial scans of the TERS signal. Nevertheless, we observed ∼1.5 times stronger TERS signal (integrating from 1100 to 1500 cm^–1^) with CW excitation from an isolated molecule adsorbed in the second monolayer (2 ML) than the SubPc molecules in the first monolayer (1 ML), as shown in Figure S10. The decay constant of the TERS signal is ∼150 pm, implying that once the tip is located on the second monolayer SubPc molecule, the TERS contribution from the 1 ML molecules is greatly reduced. Thus, the increased Raman signal from an isolated molecule in the second monolayer suggests that the TERS signal mainly comes from the single molecule underlying right below the tip. The higher signal intensity can be due to an electronic decoupling effect of the molecule from the Au substrate.^47^ Therefore, the experimentally measured nonlinear behavior of the intensity of the TERS signal upon change of the plasmonic gap suggests that single-molecule sensitivity and subnanometer lateral spatial resolution can be achieved in the case of excitation with both CW beam and ultrashort pulses.

In conclusion, we have demonstrated TERS on application of ultrashort pulses to probe various vibrational modes of a molecule. The duration of the optical pulses used in the current work, ∼500 fs, would be sufficient to probe the vibrational coherence between different modes in a molecule as well as their dynamics,^48,49^ while still preserving the spectral resolution in the TERS spectrum. The intensity of the measured TERS spectra is stable for more than 270 s, implying that the nanocavity plasmonic junction formed between the metallic tip, substrate, and molecules is stable under cryogenic temperature and UHV conditions with excitation of ultrashort pulses. The intensity of the TERS signal scales linearly with respect to the photon flux of the incident laser pulses, implying the absence of any nonlinearity in the TERS signal even with the high photon flux of the ultrashort laser pulses. The TERS signal also decays exponentially with respect to increasing the plasmonic gap size of the STM junction, indicating a very high lateral spatial resolution. Our results lay the foundation for the studies of time-resolved coherent anti-Stokes Raman spectroscopy and stimulated Raman spectroscopy on application of ultrashort pulses in the near future. This has the unique potential to track vibrational dynamics in molecules, semiconductors, and 2D materials with utmost resolutions in space, time, and energy, simultaneously.

## Supporting Information Available

Details of the local characterization of the laser pulses at the STM junction; STM image of SubPc molecules adsorbed on Au(111); TERS spectra for two different configurations of SubPc molecules (Cl up and Cl down) adsorbed on a Au(111) surface; background subtraction of the TERS spectra; spectrum of plasmonic emission from the nanocavity; tuning the central wavelength of ultrashort laser pulses with an Acousto-optical-tunable-filter (AOTF); TERS spectra excited by ultrashort laser pulses at different central wavelengths (696 and 723 nm); variation of the tunneling current as a function of increasing distance between the nanotip and a SubPc molecule adsorbed on Au(111); TERS spectra measured with a contaminated tip, when the molecule lying on the Au(111) surface gets attached to the nanotip of the STM; and comparison of TERS spectra measured from a SubPc molecule in the 1st monolayer and the 2nd monolayer (PDF)

## References

[ref1] Zewail A. H. Femtochemistry: Atomic-scale dynamics of the chemical bond. J. Phys. Chem. A 2000, 104 (24), 5660–5694. 10.1021/jp001460h. 10934390

[ref2] Krausz F.; Ivanov M. Attosecond physics. Rev. Mod. Phys. 2009, 81 (1), 163 10.1103/RevModPhys.81.163.

[ref3] Cheng Y. C.; Fleming G. R. Dynamics of light harvesting in photosynthesis. Annu. Rev. Phys. Chem. 2009, 60, 241–262. 10.1146/annurev.physchem.040808.090259. 18999996

[ref4] Nunes G.; Freeman M. R. Picosecond resolution in scanning tunneling microscopy. Science 1993, 262 (5136), 1029–1032. 10.1126/science.262.5136.1029. 17782049

[ref5] Terada Y.; Yoshida S.; Takeuchi O.; Shigekawa H. Real-space imaging of transient carrier dynamics by nanoscale pump-probe microscopy. Nat. Photonics 2010, 4 (12), 869–874. 10.1038/nphoton.2010.235.

[ref6] Cocker T. L.; Jelic V.; Gupta M.; Molesky S. J.; Burgess J. A. J.; De Los Reyes G.; Titova L. V.; Tsui Y. Y.; Freeman M. R.; Hegmann F. A. An ultrafast terahertz scanning tunnelling microscope. Nat. Photonics 2013, 7 (8), 620–625. 10.1038/nphoton.2013.151.

[ref7] Cocker T. L.; Peller D.; Yu P.; Repp J.; Huber R. Tracking the ultrafast motion of a single molecule by femtosecond orbital imaging. Nature 2016, 539 (7628), 263–267. 10.1038/nature19816. 27830788 PMC5597038

[ref8] Imada H.; Miwa K.; Imai-Imada M.; Kawahara S.; Kimura K.; Kim Y. Real-space investigation of energy transfer in heterogeneous molecular dimers. Nature 2016, 538 (7625), 364–367. 10.1038/nature19765. 27698415

[ref9] Jelic V.; Iwaszczuk K.; Nguyen P. H.; Rathje C.; Hornig G. J.; Sharum H. M.; Hoffman J. R.; Freeman M. R.; Hegmann F. A. Ultrafast terahertz control of extreme tunnel currents through single atoms on a silicon surface. Nat. Phys. 2017, 13 (6), 591–598. 10.1038/nphys4047.

[ref10] Li S. W.; Chen S. Y.; Li J.; Wu R. Q.; Ho W. Joint space-time coherent vibration driven conformational transitions in a single molecule. Phys. Rev. Lett. 2017, 119 (17), 176002 10.1103/PhysRevLett.119.176002. 29219451

[ref11] Garg M.; Kern K. Attosecond coherent manipulation of electrons in tunneling microscopy. Science 2020, 367 (6476), 411–415. 10.1126/science.aaz1098. 31727858

[ref12] Luo Y.; Jelic V.; Chen G.; Nguyen P. H.; Liu Y.-J. R.; Calzada J. A.; Mildenberger D. J.; Hegmann F. A. Nanoscale terahertz STM imaging of a metal surface. Phys. Rev. B 2020, 102 (20), 205417 10.1103/PhysRevB.102.205417.

[ref13] Müller M.; Martín-Sabanés N.; Kampfrath T.; Wolf M. Phase-resolved detection of ultrabroadband THz pulses inside a scanning tunneling microscope junction. ACS Photonics 2020, 7, 2046 10.1021/acsphotonics.0c00386. 32851116 PMC7441495

[ref14] Guo C. Y.; Meng X. Z.; Fu H. X.; Wang Q.; Wang H. M.; Tian Y.; Peng J. B.; Ma R. Z.; Weng Y. X.; Meng S.; et al. Probing nonequilibrium dynamics of photoexcited polarons on a metal-oxide surface with atomic precision. Phys. Rev. Lett. 2020, 124 (20), 206801 10.1103/PhysRevLett.124.206801. 32501065

[ref15] Garg M.; Martin-Jimenez A.; Luo Y.; Kern K. Ultrafast photon-induced tunneling microscopy. ACS Nano 2021, 15 (11), 18071–18084. 10.1021/acsnano.1c06716. PMC8613903 34723474

[ref16] Garg M.; Martin-Jimenez A.; Pisarra M.; Luo Y.; Martín F.; Kern K. Real-space subfemtosecond imaging of quantum electronic coherences in molecules. Nat. Photonics 2021, 196–202. 10.1038/s41566-021-00929-1.

[ref17] Kimura K.; Morinaga Y.; Imada H.; Katayama I.; Asakawa K.; Yoshioka K.; Kim Y.; Takeda J. Terahertz-Field-Driven Scanning Tunneling Luminescence Spectroscopy. ACS Photonics 2021, 8 (4), 982–987. 10.1021/acsphotonics.0c01755.

[ref18] Ammerman S.; Jelic V.; Wei Y.; Breslin V.; Hassan M.; Everett N.; Lee S.; Sun Q.; Pignedoli C.; Ruffieux P.; et al. Lightwave-driven scanning tunnelling spectroscopy of atomically precise graphene nanoribbons. Nat. Commun. 2021, 12 (1), 6794 10.1038/s41467-021-26656-3. 34815398 PMC8611099

[ref19] Gutzler R.; Garg M.; Ast C. R.; Kuhnke K.; Kern K. Light–matter interaction at atomic scales. Nat. Rev. Phys. 2021, 3 (6), 441–453. 10.1038/s42254-021-00306-5.

[ref20] Anderson M. S. Locally enhanced Raman spectroscopy with an atomic force microscope. Appl. Phys. Lett. 2000, 76 (21), 3130–3132. 10.1063/1.126546.

[ref21] Stöckle R. M.; Suh Y. D.; Deckert V.; Zenobi R. Nanoscale chemical analysis by tip-enhanced Raman spectroscopy. Chem. Phys. Lett. 2000, 318 (1–3), 131–136. 10.1016/S0009-2614(99)01451-7.

[ref22] Hayazawa N.; Inouye Y.; Sekkat Z.; Kawata S. Metallized tip amplification of near-field Raman scattering. Opt. Commun. 2000, 183 (1–4), 333–336. 10.1016/S0030-4018(00)00894-4.

[ref23] Domke K. F.; Zhang D.; Pettinger B. Tip-enhanced Raman spectra of picomole quantities of DNA nucleobases at Au (111). J. Am. Chem. Soc. 2007, 129 (21), 6708–6709. 10.1021/ja071107q. 17480079

[ref24] van Schrojenstein Lantman E. M.; Deckert-Gaudig T.; Mank A. J. G.; Deckert V.; Weckhuysen B. M. Catalytic processes monitored at the nanoscale with tip-enhanced Raman spectroscopy. Nat. Nanotechnol. 2012, 7 (9), 583–586. 10.1038/nnano.2012.131. 22902959

[ref25] Zhang R.; Zhang Y.; Dong Z. C.; Jiang S.; Zhang C.; Chen L. G.; Zhang L.; Liao Y.; Aizpurua J.; Luo Y.; Yang J. L.; Hou J. G. Chemical mapping of a single molecule by plasmon-enhanced Raman scattering. Nature 2013, 498 (7452), 82–86. 10.1038/nature12151. 23739426

[ref26] Jiang S.; Zhang Y.; Zhang R.; Hu C. R.; Liao M. h.; Luo Y.; Yang J. L.; Dong Z. C.; Hou J. G. Distinguishing adjacent molecules on a surface using plasmon-enhanced Raman scattering. Nat. Nanotechnol. 2015, 10 (10), 865–869. 10.1038/nnano.2015.170. 26214250

[ref27] Pozzi E. A.; Goubert G.; Chiang N.; Jiang N.; Chapman C. T.; McAnally M. O.; Henry A.-I.; Seideman T.; Schatz G. C.; Hersam M. C.; et al. Ultrahigh-vacuum tip-enhanced Raman spectroscopy. Chem. Rev. 2017, 117 (7), 4961–4982. 10.1021/acs.chemrev.6b00343. 28005348

[ref28] Wang X.; Huang S.-C.; Huang T.-X.; Su H.-S.; Zhong J.-H.; Zeng Z.-C.; Li M.-H.; Ren B. Tip-enhanced Raman spectroscopy for surfaces and interfaces. Chem. Soc. Rev. 2017, 46 (13), 4020–4041. 10.1039/C7CS00206H. 28590479

[ref29] Verma P. Tip-enhanced Raman spectroscopy: technique and recent advances. Chem. Rev. 2017, 117 (9), 6447–6466. 10.1021/acs.chemrev.6b00821. 28459149

[ref30] Sheng S.; Wu J.-b.; Cong X.; Li W.; Gou J.; Zhong Q.; Cheng P.; Tan P.-h.; Chen L.; Wu K. Vibrational properties of a monolayer silicene sheet studied by tip-enhanced Raman spectroscopy. Phys. Rev. Lett. 2017, 119 (19), 196803 10.1103/PhysRevLett.119.196803. 29219519

[ref31] Zheng L. Q.; Wang X.; Shao F.; Hegner M.; Zenobi R. Nanoscale Chemical Imaging of Reversible Photoisomerization of an Azobenzene-Thiol Self-Assembled Monolayer by Tip-Enhanced Raman Spectroscopy. Angew. Chem., Int. Ed. 2018, 130 (4), 1037–1041. 10.1002/ange.201710443. 29178528

[ref32] Whiteman P. J.; Schultz J. F.; Porach Z. D.; Chen H.; Jiang N. Dual binding configurations of subphthalocyanine on Ag (100) substrate characterized by scanning tunneling microscopy, tip-enhanced Raman spectroscopy, and density functional theory. J. Phys. Chem. C 2018, 122 (10), 5489–5495. 10.1021/acs.jpcc.7b12068.

[ref33] Kou X.; Zhou Q.; Wang D.; Yuan J.; Fang X.; Wan L. High-resolution imaging of graphene by tip-enhanced coherent anti-Stokes Raman scattering. J. Innov. Opt. Health Sci. 2019, 12 (01), 1841003 10.1142/S1793545818410031.

[ref34] Lee J.; Crampton K. T.; Tallarida N.; Apkarian V. A. Visualizing vibrational normal modes of a single molecule with atomically confined light. Nature 2019, 568 (7750), 78–82. 10.1038/s41586-019-1059-9. 30944493

[ref35] Zhang Y.; Yang B.; Ghafoor A.; Zhang Y.; Zhang Y. F.; Wang R. P.; Yang J. L.; Luo Y.; Dong Z. C.; Hou J. G. Visually constructing the chemical structure of a single molecule by scanning Raman picoscopy. Natl. Sci. Rev. 2019, 6 (6), 1169–1175. 10.1093/nsr/nwz180. 34691995 PMC8291412

[ref36] Jaculbia R. B.; Imada H.; Miwa K.; Iwasa T.; Takenaka M.; Yang B.; Kazuma E.; Hayazawa N.; Taketsugu T.; Kim Y. Single-molecule resonance Raman effect in a plasmonic nanocavity. Nat. Nanotechnol. 2020, 15 (2), 105–110. 10.1038/s41565-019-0614-8. 31959928

[ref37] Xu J.; Zhu X.; Tan S.; Zhang Y.; Li B.; Tian Y.; Shan H.; Cui X.; Zhao A.; Dong Z.; et al. Determining structural and chemical heterogeneities of surface species at the single-bond limit. Science 2021, 371 (6531), 818–822. 10.1126/science.abd1827. 33602852

[ref38] Wang R.-P.; Yang B.; Fu Q.; Zhang Y.; Zhu R.; Dong X.-R.; Zhang Y.; Wang B.; Yang J.-L.; Luo Y.; et al. Raman Detection of Bond Breaking and Making of a Chemisorbed Up-Standing Single Molecule at Single-Bond Level. J. Phys. Chem. Lett. 2021, 12 (7), 1961–1968. 10.1021/acs.jpclett.1c00074. 33591760

[ref39] Liu S.; Hammud A.; Wolf M.; Kumagai T. Atomic Point Contact Raman Spectroscopy of a Si (111)-7× 7 Surface. Nano Lett. 2021, 21 (9), 4057–4061. 10.1021/acs.nanolett.1c00998. 33934600 PMC8288640

[ref40] Li L.; Schultz J. F.; Mahapatra S.; Liu X.; Shaw C.; Zhang X.; Hersam M. C.; Jiang N. Angstrom-Scale Spectroscopic Visualization of Interfacial Interactions in an Organic/Borophene Vertical Heterostructure. J. Am. Chem. Soc. 2021, 143 (38), 15624–15634. 10.1021/jacs.1c04380. 34369773

[ref41] Lee J.; Tallarida N.; Chen X.; Liu P.; Jensen L.; Apkarian V. A. Tip-enhanced Raman spectromicroscopy of Co (II)-tetraphenylporphyrin on Au (111): Toward the chemists’ microscope. ACS Nano 2017, 11 (11), 11466–11474. 10.1021/acsnano.7b06183. 28976729

[ref42] Klingsporn J. M.; Sonntag M. D.; Seideman T.; Van Duyne R. P. Tip-enhanced Raman spectroscopy with picosecond pulses. J. Phys. Chem. Lett. 2014, 5 (1), 106–110. 10.1021/jz4024404. 26276188

[ref43] Pozzi E. A.; Sonntag M. D.; Jiang N.; Chiang N.; Seideman T.; Hersam M. C.; Van Duyne R. P. Ultrahigh vacuum tip-enhanced Raman spectroscopy with picosecond excitation. J. Phys. Chem. Lett. 2014, 5 (15), 2657–2661. 10.1021/jz501239z. 26277959

[ref44] Neese F. The ORCA program system. Wiley Interdiscip. Rev. Comput. 2012, 2 (1), 73–78. 10.1002/wcms.81.

[ref45] Weigend F.; Ahlrichs R. Balanced basis sets of split valence, triple zeta valence and quadruple zeta valence quality for H to Rn: Design and assessment of accuracy. Phys. Chem. Chem. Phys. 2005, 7 (18), 3297–3305. 10.1039/b508541a. 16240044

[ref46] Grimme S.; Antony J.; Ehrlich S.; Krieg H. A consistent and accurate ab initio parametrization of density functional dispersion correction (DFT-D) for the 94 elements H-Pu. J. Chem. Phys. 2010, 132 (15), 154104 10.1063/1.3382344. 20423165

[ref47] Chen G.; Zhu J.; Li X. Influence of a dielectric decoupling layer on the local electric field and molecular spectroscopy in plasmonic nanocavities: a numerical study. Chin. Opt. Lett. 2021, 19 (12), 123001 10.3788/COL202119.123001.

[ref48] Voronine D. V.; Sinyukov A. M.; Hua X.; Wang K.; Jha P. K.; Munusamy E.; Wheeler S. E.; Welch G.; Sokolov A. V.; Scully M. O. Time-resolved surface-enhanced coherent sensing of nanoscale molecular complexes. Sci. Rep. 2012, 2 (1), 891 10.1038/srep00891. 23189240 PMC3506977

[ref49] Yampolsky S.; Fishman D. A.; Dey S.; Hulkko E.; Banik M.; Potma E. O.; Apkarian V. A. Seeing a single molecule vibrate through time-resolved coherent anti-Stokes Raman scattering. Nat. Photonics 2014, 8 (8), 650–656. 10.1038/nphoton.2014.143.

