# Supplementary material for: Ultrashort Pulse Excited Tip-Enhanced Raman Spectroscopy in Molecules
Source: Nano Lett. 2022 Jun 15;22(13):5100–6. doi: 10.1021/acs.nanolett.2c00485 (PMC9284611; doi:10.1021/acs.nanolett.2c00485)
Supplement: Supplementary file 1 — nl2c00485_si_001.pdf [file nl2c00485_si_001.pdf]

Supplementary Information

Ultrashort Pulse Excited Tip-Enhanced Raman Spectroscopy in Molecules

Yang Luo[1], Alberto Martin-Jimenez[1], Rico Gutzler[1], Manish Garg[1*], Klaus Kern[1, 2]

[1] Max Planck Institute for Solid State Research, Heisenbergstr. 1, 70569 Stuttgart, Germany

[2] Institut de Physique, Ecole Polytechnique Fédérale de Lausanne, 1015 Lausanne, Switzerland

[*]Author to whom correspondence should be addressed.

Email: mgarg@fkf.mpg.de

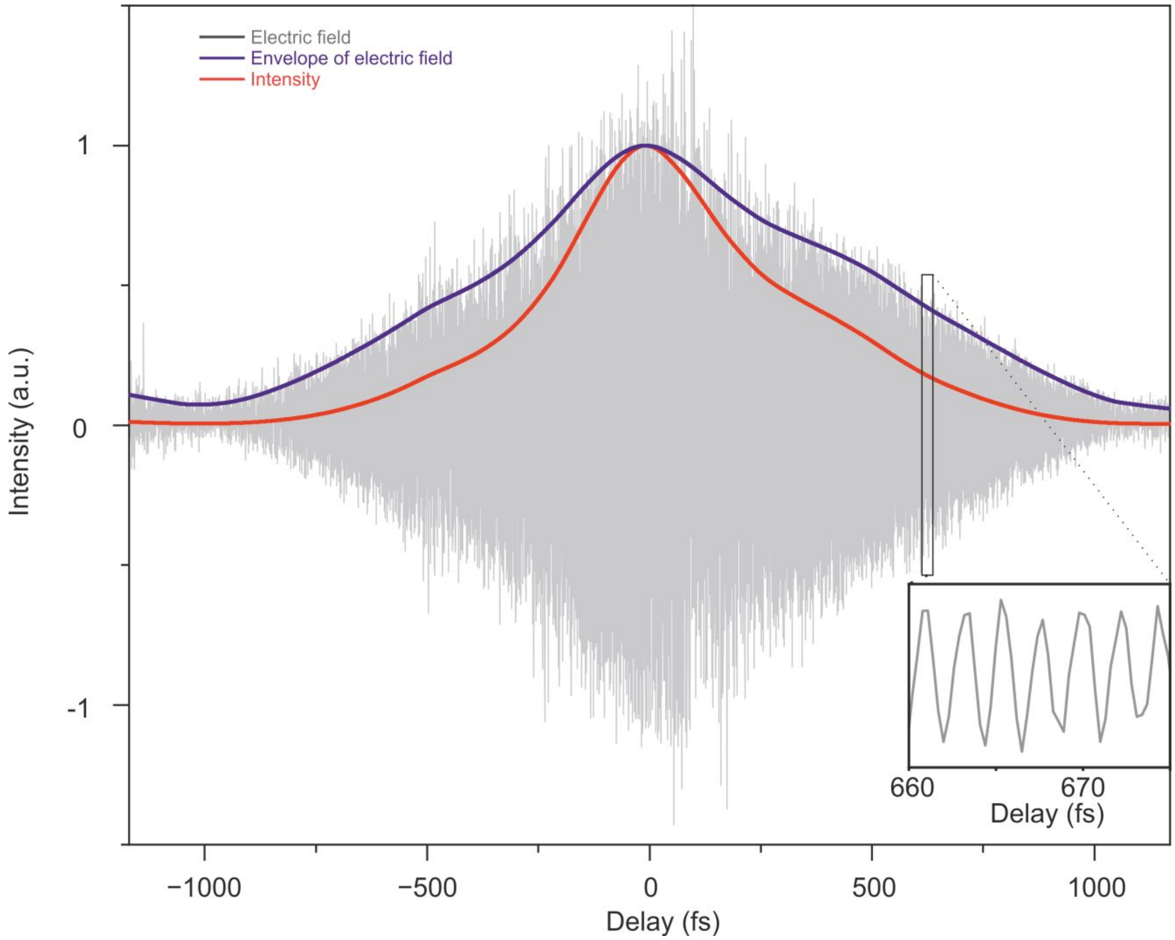

Figure S1. Characterization of the ultrashort pulses at the STM junction by the technique of homodyne beating. A detailed description of the homodyne beating technique can be found in our earlier works in Ref. [1,2] The grey curve represents the measured electric field of the ultrashort pulses, **E(t)**, which oscillates with the time period of ~ 2.4 fs, consistent with the wavelength of 728 nm. The violet curve shows the envelope of the electric field. The temporal intensity profile ($I(t) = |\mathbf{E(t)}|^2$) of the laser pulse is shown by the red curve. Inset shows the oscillation of the wave cycles of the electric filed of the ultrashort pulses in a narrow time window.

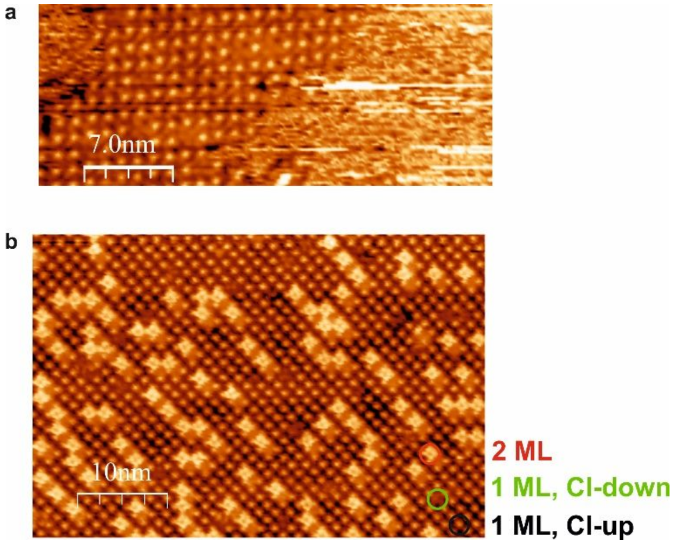

Figure S2. (a) STM image of SubPc molecules on Au(111) with a coverage below one monolayer. (b) STM image of SubPc molecules displaying full monolayer coverage and few second monolayer molecules on Au(111). Black and green circles mark the positions of two adjacent SubPc molecules with opposite orientations of the Cl atoms. The red circle shows an isolated SubPc molecule in the 2nd monolayer. When the coverage is below 1 monolayer, diffusion of the molecules at 90 K prevents forming self assembled islands, resulting in a fuzzy imaging. When the coverage is above 1 monolayer, a complete monolayer of SubPc molecules is formed, and isolated SubPc molecules in the 2nd monolayer can also be seen. We do not observe islands where SubPc molecules are either in Cl up or Cl down configurations entirely.

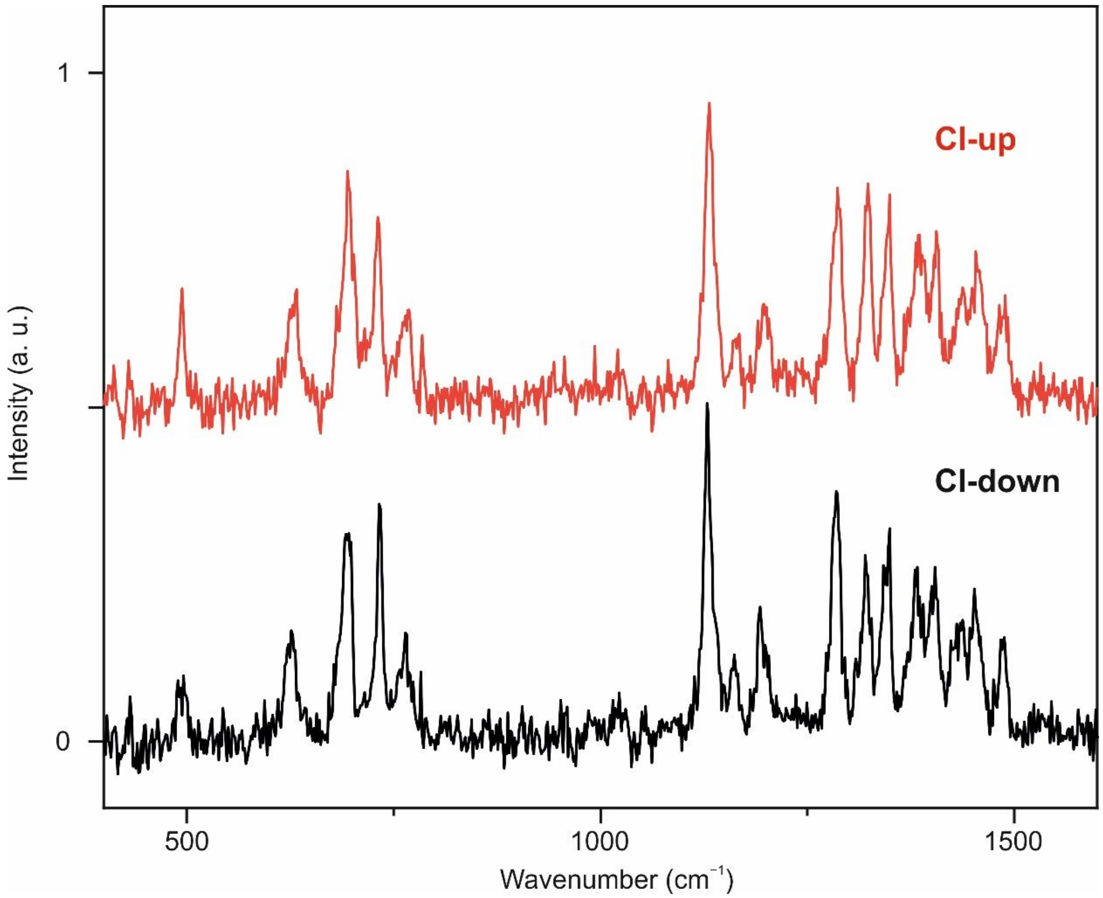

Figure S3. Comparison of the TERS spectra when the STM nanotip is placed on top a SubPc molecule where the Cl atom points up (red curve) and where the Cl atom points down (black curve). The spectra were acquired with CW excitation (P = 1.1 mW, V = 100 mV, I = 1 nA, t = 30 s).

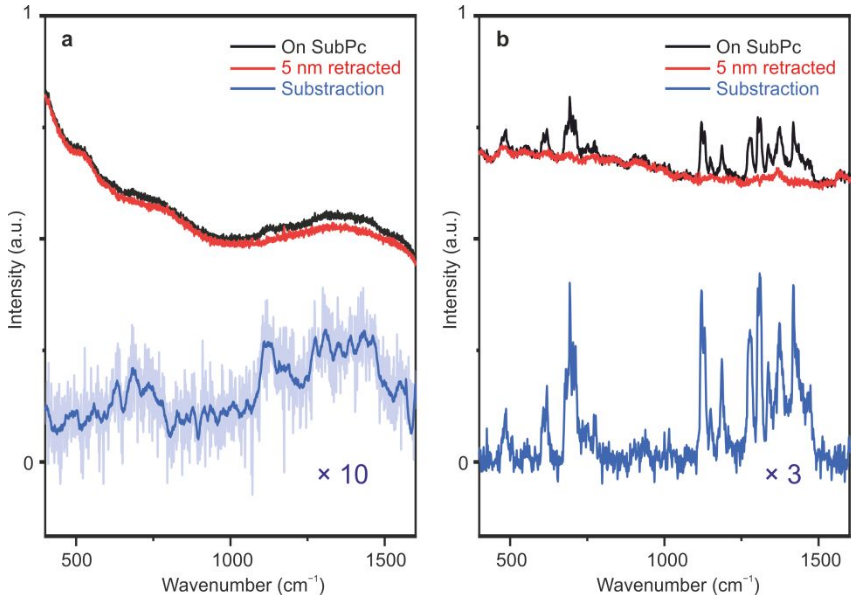

Figure S4. TERS spectra acquired with ultrashort pulse (a) and with CW laser (b) excitation. The Raman spectra (blue curves) were obtained by subtracting the spectra recorded on a SubPc molecule with the nanotip being in tunneling contact (black curves) from the spectra recorded when the tip is retracted by ~ 5 nm from the Au(111) surface (red curves).

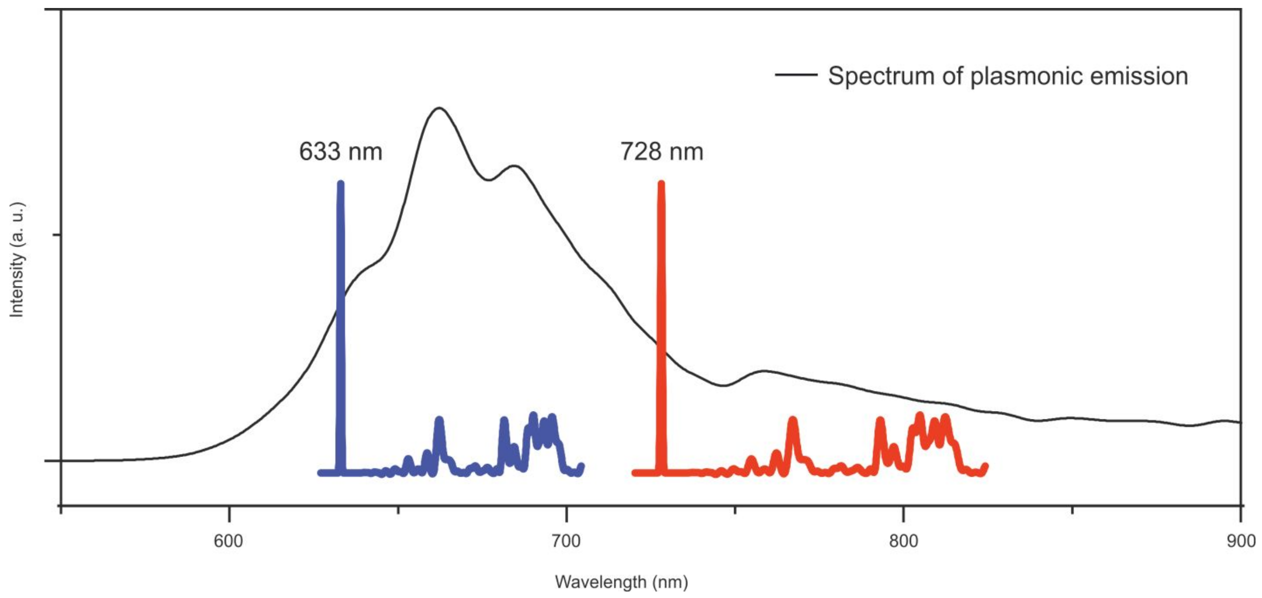

Figure S5. The experimentally measured plasmonic emission spectrum from the nanocavity is shown by the black curve. The spectrum was acquired (acquisition time 30 s) with a bias voltage of 3V and a set point current of 8 nA. The blue and red curves indicate the peak positions of the Raman signal at the excitation wavelengths of 633 nm and 728 nm, respectively.

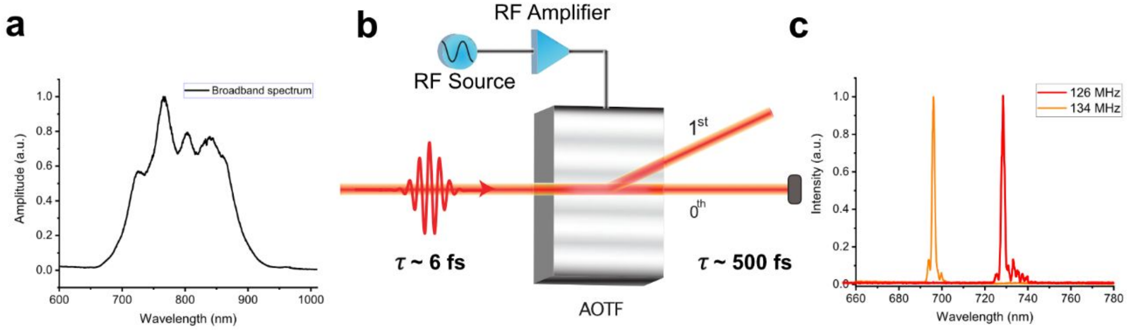

Figure S6. (a) Spectrum of the ultrashort broadband laser pulses being focused to an Acousto-optical-tunable-filter (AOTF). (b) AOTF operating with a tunable RF waveform generator and a RF amplifier (Power < 2 W). **c**, Spectra of the 1st order diffracted beam out of the AOTF for two different driving frequencies as mentioned in the legend. The central wavelength of the ~ 500 fs long laser pulse is around ~ 696 nm and ~723 nm for driving frequencies of 134 MHz and 128 MHz, respectively.

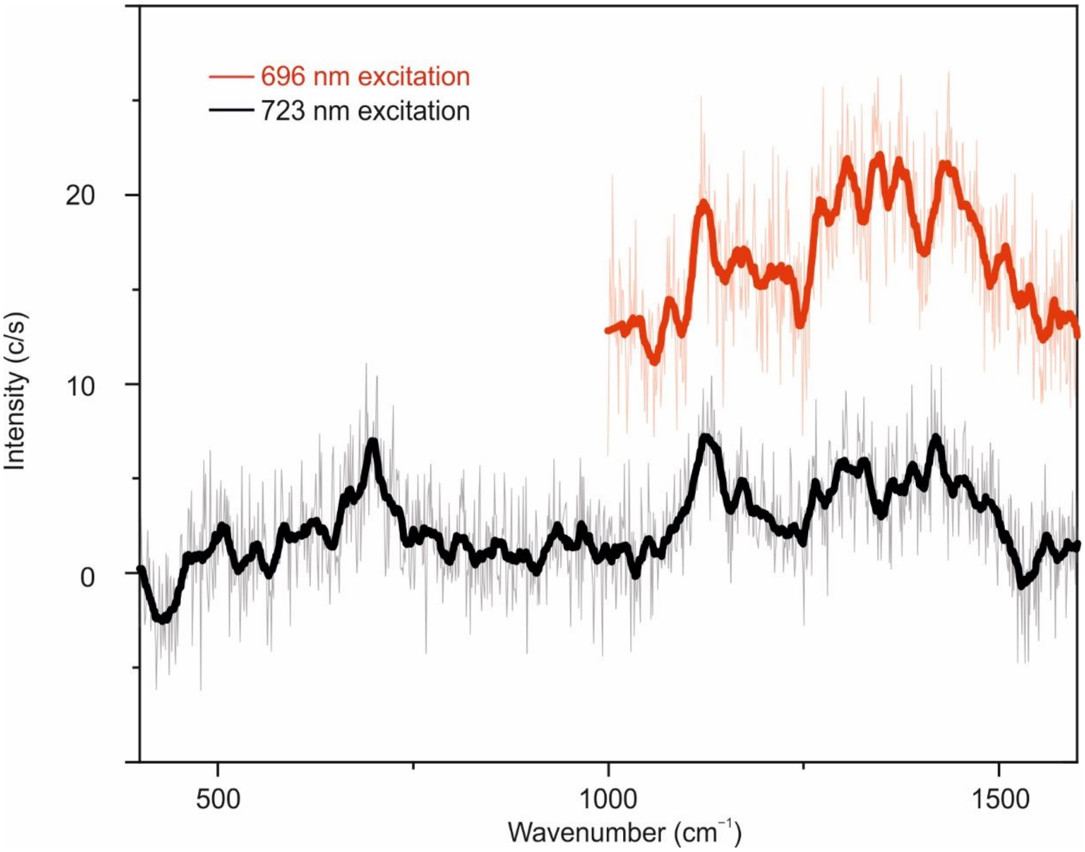

Figure S7. Comparison of the TERS spectra excited by ultrashort laser pulses at different central wavelengths, 696 nm (red curve) and 723 nm (black curve). The laser power is 1 mW in both measurements. We used a 740 nm long-pass dichroic mirror in the experiments, thus the signal below 1000 cm^{-1} generated with 696-nm excitation is not measured. In the TERS spectrum acquired with the laser pulses centered at 696 nm, the Raman intensity is ~ 50% stronger (integrating from 1100 cm^{-1} to 1500 cm^{-1}) than that of the 723-nm pulses. This is because the wavelength of 696 nm is closer to the peak position of plasmonic emission from the STM nanocavity. The combination of AOTF and broadband laser pulses allows for continuous tuning of the excitation wavelength of the ultrashort laser pulses for TERS measurements.

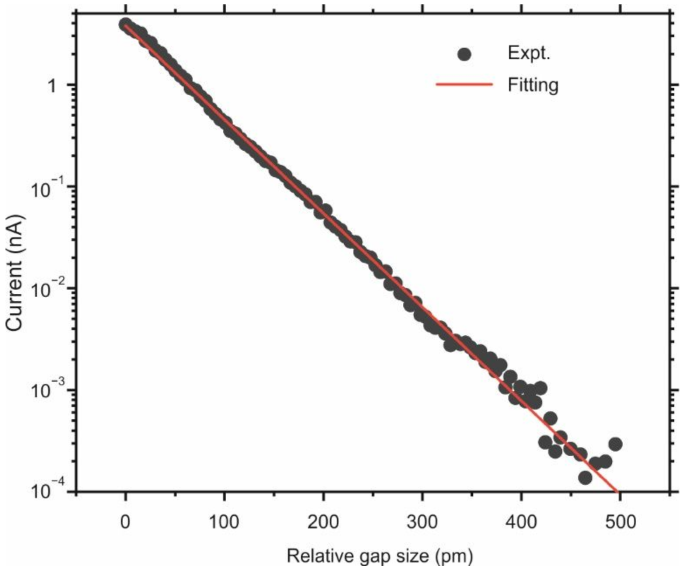

Figure S8. Variation of the tunneling current (I) as a function of increasing distance (Δz) between the nanotip and a SubPc molecule adsorbed on Au(111). From the fitting of the I-Δz curve (red curve), we can obtain the apparent barrier height (ABH) following the equation $\phi = \frac{\mathrm{h}^2}{8m} \cdot \left(\frac{d \ln I}{dz} \right)^2$, where m is the electron mass. Here, we measured an ABH of 4.3 eV, which is similar to the value that one would obtain on a clean Au(111) surface. This suggests that a molecular layer on top of Au(111) would not alter the behavior of the I-Δz curve.

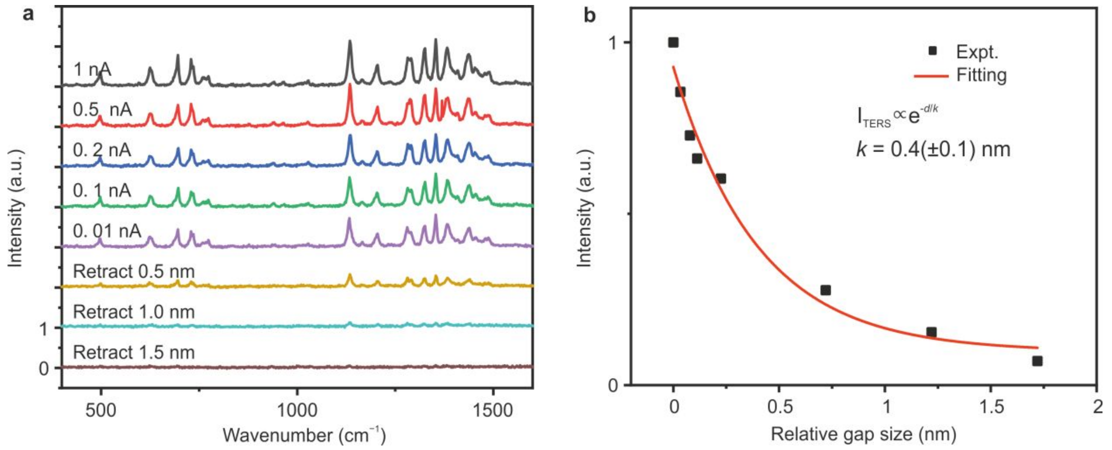

Figure S9. (a) Series of CW-TERS spectra acquired as a function of increasing tunneling current (decreasing plasmonic gap size) at the STM junction. The spectra were acquired for an incident laser power of 1 mW, with a bias voltage of 100 mV at the STM junction. The acquisition time of each spectrum was 30 s. (b) Variation of the integrated intensity of the CW-TERS signal with the plasmonic gap size.

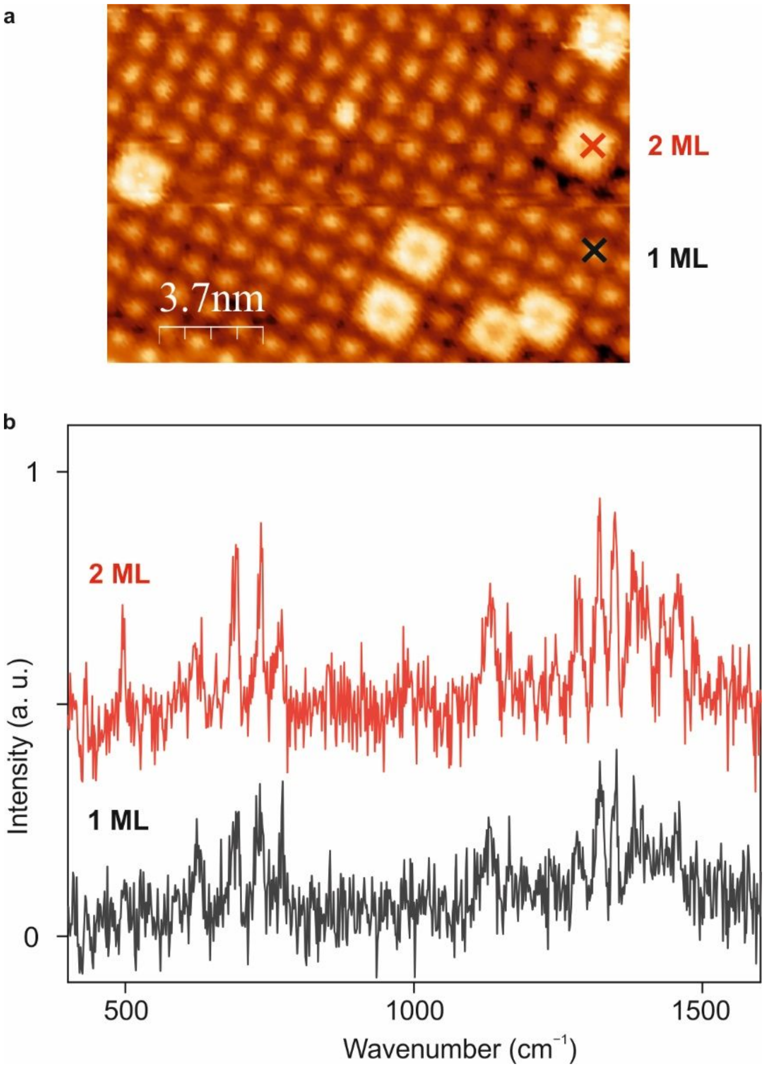

Figure S10. (a) STM image showing isolated SubPc molecules in the 2nd monolayer on top of a complete monolayer coverage of SubPc molecules on top of Au(111). (b) Comparison of the TERS spectra measured when placing the STM nanotip on top of an isolated molecule in the 2nd monolayer (red-curve, vertically shifted for clarity) and on top of a molecule in the 1st monolayer (black-curve). The position of the STM nanotip in the two measurements is marked by red (isolated molecule) and black (molecule in 1st monolayer) crosses. The spectra were acquired with CW excitation (P = 1.1 mW, V = 100 mV, I = 0.2 nA, t = 30 s).

References:

1 Garg, M., Martin-Jimenez, A., Luo, Y. & Kern, K. Ultrafast photon-induced tunneling microscopy. *ACS Nano* **15**, 18071-18084 (2021).

2 Garg, M. *et al.* Real-space subfemtosecond imaging of quantum electronic coherences in molecules. *Nat. Photonics*, 1-7 (2021).
